# Caregiving Profiles of Family Members Assisting Older Mexican American Adults with Varying Care Needs

**DOI:** 10.1093/geroni/igaf122.2646

**Published:** 2025-12-31

**Authors:** Karen Schlag, Soham Al Snih, Monique Pappadis

**Affiliations:** University of Texas Medical Branch at Galveston, Galveston, Texas, United States; The University of Texas Medical Branch at Galveston, Texas, Galveston, Texas, United States; University of Texas Medical Branch at Galveston, Galveston, Texas, United States

## Abstract

This study analyzed population-based data to identify caregiving profiles observed among Mexican American adults who assist older relatives with varying levels of physical and cognitive limitations. Using data from the from the 2016 Hispanic Established Populations for the Epidemiological Study of the Elderly (H-EPESE) Wave 9 Caregiver supplement (n = 460), we conducted latent class analysis in Stata to estimate latent profiles of family caregivers. Indicators for analysis included 9 variables relaying degree of caregiving responsibility types (i.e., personal care, household tasks, medical care, financial support), patient conditions (i.e., needs assistance with activities of daily living, cognitive impairment), family dynamics (i.e., lives with care recipient, degree of conflict with them) and use of formal support services. Results supported a typology of three discernable family caregiving profiles: 1.) intense caregiving (43% of sample, e.g., high caregiving responsibility and care recipient need, likely to live with care recipient, moderate formal support); 2.) low demand caregiving (37% of the sample, minimal caregiving responsibility, moderate care recipient need, moderately likely to live with care recipient, low formal support); 3.) moderate caregiving (20% of sample, e.g., moderate caregiving responsibility, high care recipient need, less likely to live with care recipient, moderate formal support. Findings will inform a future study to investigate the associations between distinct caregiving profiles and caregiver health outcomes among family members of older Mexican American adults.

